# Curative Treatment of Candidiasis by the Live Biotherapeutic Microorganism *Lactobacillus rhamnosus* Lcr35^®^ in the Invertebrate Model *Caenorhabditis elegans*: First Mechanistic Insights

**DOI:** 10.3390/microorganisms8010034

**Published:** 2019-12-23

**Authors:** Cyril Poupet, Philippe Veisseire, Muriel Bonnet, Olivier Camarès, Marylise Gachinat, Caroline Dausset, Christophe Chassard, Adrien Nivoliez, Stéphanie Bornes

**Affiliations:** 1 Université Clermont Auvergne, INRAE, VetAgro Sup, UMRF, 15000 Aurillac, France; 2 Biose Industrie, 24 avenue Georges Pompidou, 15000 Aurillac, France

**Keywords:** *Lactobacillus rhamnosus* Lcr35^®^, *Candida albicans*, *Caenorhabditis elegans*, curative treatment, immune response

## Abstract

The resistance of *Candida albicans* to conventional drug treatments, as well as the recurrence phenomena due to dysbiosis caused by antifungal treatments, have highlighted the need to implement new therapeutic methodologies. The antifungal potential of live biotherapeutic products (LBP) has already been demonstrated using preclinical models (cell cultures, laboratory animals). Understanding their mechanisms of action is strategic for the development of new therapeutics for humans. In this study, we investigated the curative anti-*C. albicans* properties of *Lactobacillus rhamnosus* Lcr35^®^ using the in vitro Caco-2 cell and the in vivo *Caenorhabditis elegans* models. We showed that Lcr35^®^ does inhibit neither the growth (*p* = 0.603) nor the biofilm formation (*p* = 0.869) of *C. albicans* in vitro. Lcr35^®^ protects the animal from the fungal infection (+225% of survival, *p* < 2 × 10^–16^) even if the yeast is detectable in its intestine. In contrast, the Lcr35^®^ cell-free supernatant does not appear to have any antipathogenic effect. At the mechanistic level, the DAF-16/Forkhead Box O transcription factor is activated by Lcr35^®^ and genes of the p38 MAP Kinase signaling pathway and genes involved in the antifungal response are upregulated in presence of Lcr35^®^ after *C. albicans* infection. These results suggest that the LBM strain acts by stimulating its host via DAF-16 and the p38 MAPK pathway.

## 1. Introduction

Despite being a minority in relation to bacterial infections, fungal infections are a growing public health problem. The list of opportunistic fungi causing serious, life-threatening fungal diseases increases almost every year and contains species belonging to *Candida*, *Aspergillus*, *Cryptococcus*, *Geotrichum*, *Trichosporon*, *Rhodotorula*, or *Saccharomyces* genera [1,2].

Yeasts of the genus *Candida* are frequent colonizers of the skin and mucous membranes of animals and dissemination in nature is widespread. Only a few of the more than 150 described species are regularly found as infectious agents in humans including *C. albicans*, in over 50% of cases [2]. The term candidiasis comprises several categories of infections: systemic or invasive *Candida* infections/diseases, such as candidaemia as well as superficial *Candida* infections [3]. Beyond the systemic manifestations, *C. albicans* is also the cause of infection of the gastrointestinal and vaginal tracts (vulvovaginal candidiasis) [4,5]. The candidiasis and fungal infections in general are becoming more difficult to treat, mainly because of the emergence of antimicrobial multiresistances [6]. This is particularly associated with a decrease in the quality of life (state of health) of patients and a very strong increase in associated medical costs [7].

The concept of anti-infective or antifungal stewardship (AFS) may be defined as an ongoing healthcare effort to optimize antimicrobial use in order to improve patient outcomes, ensure cost-effective therapy and limit antimicrobial resistance [8]. Concepts may not only include the appropriate use of antimicrobials by selecting the proper drug, dosage, duration, route of administration and finally costs related to patient’s management. An understanding of the pharmacokinetics and pharmacodynamics (PK/PD) of these drugs has been demonstrated to be important to optimize drug choice and dosing regimen [2].

One of the alternatives to the use of traditional antifungals could be the establishment of a therapy with Live Biotherapeutic Products (LBP). A LBP, as defined by the Center for Biologics Evaluation and Research (CBER), is “a biological product that: (1) contains live organisms, such as bacteria; (2) is applicable to the prevention, treatment, or cure of a disease or condition of human beings; and (3) is not a vaccine” [9]. Described in the literature for many years, they have the status of Generally Regarded as Safe (GRAS) in the United States of America and Qualified Presumption of Safety (QPS) according to European Food Safety Authority (EFSA) because of the absence of adverse effects when consumed in humans [10]. The interest in using Live Biotherapeutic Microorganisms lies in two characteristics. They initially allow the inhibition and elimination of the pathogen(s) and in a second time, the restoration of a healthy commensal microbiota.

During in vitro investigations, Nivoliez et al. [11] demonstrated the ability of *Lactobacillus rhamnosus* Lcr35^®^ to kill the pathogenic yeast *Candida albicans* and also *Gardnerella vaginalis*, frequently encountered during bacterial vaginosis and some intestinal pathogens as *Escherichia coli* (EPEC, ETEC) and *Shigella flexnerii*. Moreover, beyond the anti-pathogenic properties, its weak persistence on the mucous membranes linked to its transient residency on mucous membranes is favorable to the resilience of the commensal microbiota. Up to now, we know little about the molecular mechanisms underlying these anti-pathogen properties. Deciphering these mechanisms therefore requires the use of cellular and animal models having enough homology with humans. This type of approach, implemented during our previous work [12], highlighted the preventive protective capacities of the LBM on intestinal Caco-2 cells and the invertebrate *Caenorhabditis elegans*, with respectively an inhibition of the growth of the yeast and an optimal survival of the host. So far, most studies have been conducted in contexts of fungal infection prevention. These have provided answers about the mechanisms of action allowing an LBM to prevent the installation of a pathogen with, for instance, the reduction of *C. albicans* hyphae formation [12,13,14,15,16]. However, few studies showing the efficacy of LBM against candidiasis have been conducted and no “curative” study relative to *C. albicans* was conducted using the nematode *C. elegans* [12,16]. The only curative study was carried out by Sharma and colleagues against infections with *E. coli* pathogenic strains [17]. In addition, it is important to note that patients rarely take preventive treatment instead of a curative one. The study presented here is the first in vitro and in vivo use of an LBM to curatively treat candidiasis.

With this aim in view, we proposed two experimental models to study the host-microorganisms and microorganism-microorganism interactions. In this context, we wanted to evaluate the effect of *Lactobacillus rhamnosus* Lcr35^®^ strain to cure a fungal infection due to *C. albicans.* In order to overcome the experimental limits of the Caco-2, an enterocyte-like cell line in vitro model, we conducted the mechanistic study on *C. elegans,* an in vivo model relevant for interaction and mechanistic investigations [12]. The worm survival and gene expression, in response to the pathogen and/or the LBM, were evaluated.

## 2. Results

### 2.1. Anti C. albicans Effects of L. rhamnosus Lcr35^®^ on Caco-2 Cell Monolayer

#### 2.1.1. Growth Inhibition of *C. albicans*

**Figure 1:**
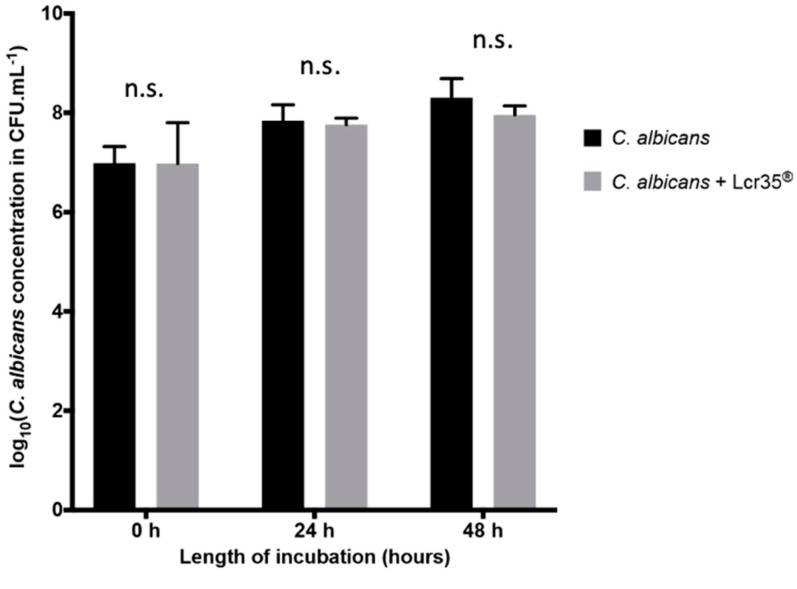
Evolution of the concentration of *C. albicans* in the presence or not of Lcr35^®^ onto Caco-2 cells monolayers. Results are expressed as log_10_ CFU/mL of yeasts alone (controls) and in co-incubation with Lcr35^®^ (mean ± standard deviation). Comparison between conditions with and without Lcr35^®^ was performed using a two-way ANOVA followed by a Fisher’s Least Significant Difference (LSD) post hoc test (n.s.: not significant).

On a Caco-2 cells monolayer, the yeast grew to 8.31 ± 0.38 log CFU/mL after 48 h of incubation. With a Lcr35^®^ curative treatment, we observed a similar growth of the yeast which reached 7.96 ± 0.18 log CFU/mL (Figure 1). Thus, no significant inhibition (after 24 h, *p* = 0.902; after 48 h, *p* = 0.603) of yeast growth was noted in the presence of the LBM in vitro and in curative condition.

#### 2.1.2. Inhibition of *C. albicans* Biofilm Formation

**Figure 2:**
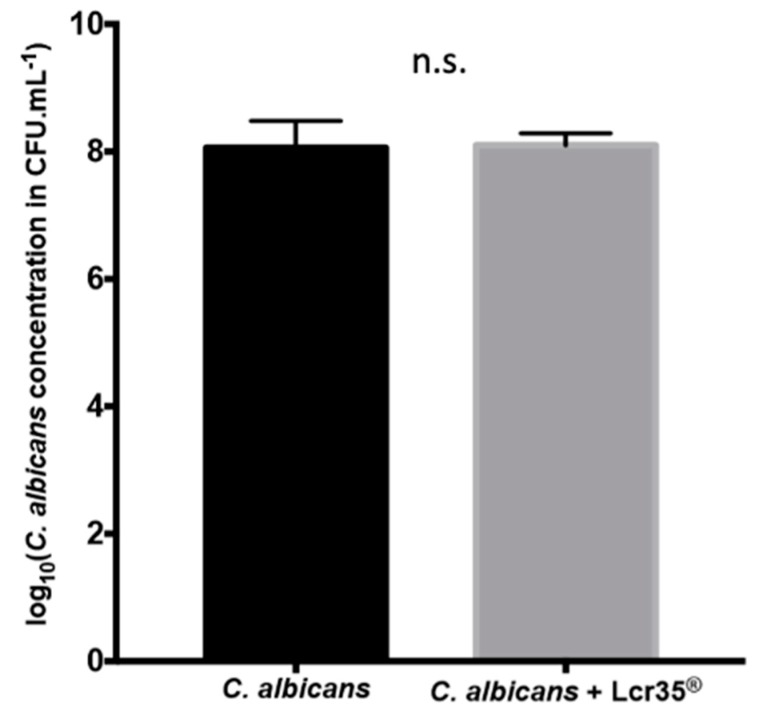
Determination of *C. albicans* concentration present in the biofilm in presence of Lcr35^®^ or not onto Caco-2 cells monolayers (mean ± standard deviation). The amount of yeast present in the biofilm was evaluated after 48 h of incubation. Comparison between conditions with and without Lcr35^®^ was performed using an unpaired t test (n.s.: not significant).

After 48 h of incubation, the *C. albicans* biofilm contained 8.06 ± 0.42 log CFU/mL of yeasts. A similar concentration has been measured after a Lcr35^®^ curative treatment with 8.10 ± 0.19 log CFU/mL of yeasts (Figure 2). These results suggest that Lcr35^®^ does not inhibit (*p* = 0.869) the formation of a biofilm by *C. albicans* in vitro.

### 2.2. Effect of L. rhamnosus Lcr35^®^ Curative Treatment on Candidiasis

#### 2.2.1. Effect of *L. rhamnosus* Lcr35^®^ on *C. elegans* Survival after *C. albicans* Exposure

**Figure 3:**
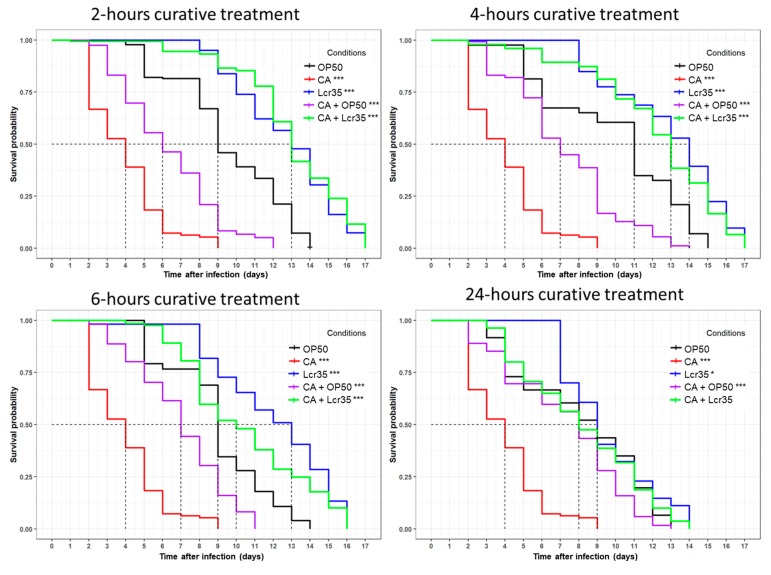
Curative effects of Lcr35^®^ against *C. albicans* ATCC 10231. Mean survival, where half of the population is dead, is represented on the abscissa. The asterisks indicate the *p*-values (log-rank test) against OP50 (*p* <0.05: *; *p* <0.001: ***). Infection duration: 2 h; 2-h curative treatment (*E. coli* OP50 (OP50, *n* = 179); *C. albicans* ATCC 10231 (CA, *n* = 424); Lcr35^®^ (Lcr35, *n* = 161); *E. coli* OP50 + *C. albicans* (OP50 + CA, *n* = 119); Lcr35^®^ + *C. albicans* (Lcr35 + CA, *n* = 163)); 4-h curative treatment (*E. coli* OP50 (OP50, *n* = 143); *C. albicans* ATCC 10231 (CA, *n* = 424); Lcr35^®^ (Lcr35, *n* = 259); *E. coli* OP50 + *C. albicans* (OP50 + CA, *n* = 274); Lcr35^®^ + *C. albicans* (Lcr35 + CA, *n* = 198)); 6-h curative treatment (*E. coli* OP50 (OP50, *n* = 222); *C. albicans* ATCC 10231 (CA, *n* = 424); Lcr35^®^ (Lcr35, *n* = 165); *E. coli* OP50 + *C. albicans* (OP50 + CA, *n* = 293); Lcr35^®^ + *C. albicans* (Lcr35 + CA, *n* = 129)); 24-h curative treatment (*E. coli* OP50 (OP50, *n* = 148); *C. albicans* ATCC 10231 (CA, *n* = 424); Lcr35^®^ (*n* = 170); *E. coli* OP50 + *C. albicans* (OP50 + CA, *n* = 290); Lcr35^®^ + *C. albicans* (Lcr35 + CA, *n* = 160)).

When *C. elegans* was sequentially infected by *C. albicans* for 2 h prior being exposed for 2 h to Lcr35^®^, the survival of the nematodes was significantly increased as the mean lifespan rose from 4 to 13 days (225% increase) compared with that observed with *C. albicans* infection alone (*p* < 2 × 10^–16^) (Figure 3). There was no significant difference between worms sequentially exposed to Lcr35^®^ and *C. albicans* and those exposed to Lcr35^®^ only (*p* = 0.2). Similar results were obtained when the nematodes were exposed to the LBM for 4 h. In this case, we observed that Lcr35^®^ protected *C. elegans* from infection even if we observed a slight difference with the Lcr35^®^ control condition without infection (*p* = 0.04). We note that the difference of *C. elegans* lifespan between the 2-h and 4-h curative treatments with Lcr35^®^ was not statistically significant (*p* = 0.06).

For longer Lcr35^®^ treatment times (6 and 24 h), we observed a significant decrease of *C. elegans* mean survival on Lcr35^®^ alone (condition 6 h: *p* = 3 × 10^–4^, condition 24 h: *p* < 2 × 10^–16^) or in the presence of both Lcr35^®^ and *C. albicans* (condition 6 h: *p* = 5 × 10^–6^, condition 24 h: *p* < 2 × 10^–16^) compared to the treatment of 4 h. Taken together, the results showed that the 2-h and the 4-h LBM treatments were the most protective against *C. albicans* infection.

#### 2.2.2. Influence of *L. rhamnosus* Lcr35^®^ on *C. albicans* Presence in the Worm Gut

**Figure 4:**
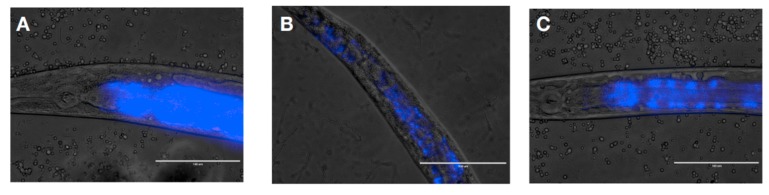
*C. albicans* colonization of *C. elegans* gut after 72 h (**A**) and after a 24-h-curative treatment with *E. coli* OP50 (**B**) or Lcr35^®^ (**C**). The blue color represents yeast labeled with DAPI. Scale bar = 10 μm.

In order to determine whether the anti-*Candida* effects observed were due to the removal of the pathogen, colonization of the intestine of the nematode by *C. albicans* was observed by light microscopy. After three days of incubation in presence of the yeast, the digestive tract of the worm was fully colonized (Figure 4A). We subsequently applied a curative treatment to the worms for 24 h. We observed that after a curative treatment with the control OP50 (Figure 4B) or the LBM Lcr35^®^ (Figure 4C), the yeast *C. albicans* was still detected in the digestive tract of the host.

### 2.3. Effect of L. rhamnosus Lcr35^®^ Cell-Free Supernatant on Candidiasis

The objective of this experiment was to determine whether the anti-*C. albicans* properties of Lcr35^®^ were due to the production of metabolites in the culture supernatant (Table 1). When the worms were previously fed with *E. coli* OP50 and then placed in the presence of 30 or 50% of Lcr35^®^ supernatant, we observed a significant increase (*p* < 2 × 10^–16^) of their mean lifespan and longevity compared to the control condition (*E. coli* OP50 strain without supernatant). We note that there was no significant difference (*p* = 0.2) between the control condition Lcr35^®^ and the *E. coli* OP50 condition supplemented with 30% supernatant at pH = 7. Since the worms were in the presence of undiluted supernatant (i.e., 100%), we observed a very strong decrease (*p* < 2 × 10^–16^) of median survival and longevity, respectively equal to 1 and 8 days. This result was obtained for both the supernatant at native pH (pH = 4.5) and the neutralized supernatant (pH = 7).

In the case of the worms infected with *Candida albicans* and then treated using cell-free supernatant, only the concentration 30% at pH 4.5 induced a better resistance of the host to the infection (*p* < 2 × 10–16). Indeed, as soon as the concentration of the supernatant was increased (50 or 100%) or its pH was neutralized, we observed a decrease (*p* < 2 × 10–16) of the survival of the nematode, which under certain conditions, was lower compared to the control condition *C. albicans*.

### 2.4. Mechanistic Study

In order to explain the mechanisms of action underlying the curative protection of *L. rhamnosus* Lcr35^®^ in the nematode, a mechanistic approach was implemented by studying the modulation of the expression of genes of interest and the cellular localization of the transcription factor DAF-16 in the host. A search for the presence of yeast in the digestive tract was also performed. Due to the fact that the culture supernatant of Lcr35^®^ does not have anti-*Candida* effect in the nematode, the mechanistic study on this aspect was not performed.

#### 2.4.1. Modulation of *C. elegans* Genes Expression Induced by Lcr35^®^ and *C. albicans*

To elucidate the mechanisms involved in the action of Lcr35^®^ against *C. albicans*, we studied the expression of seven *C. elegans* genes (Table 2) divided into three groups: *daf-2* and *daf-16* (insulin signaling pathway) involved in host longevity and antipathogenicity response, *sek-1* and *pmk-1* (p38 MAPK signaling pathway) which concern the immunity reaction as well as *abf-2*, *cnc-4* and *fipr-22*/*fipr-23* which encode antimicrobial proteins. The interest in targeting these genes has been demonstrated by various studies showing that the insulin and p38 MAPK pathways were involved in the pro-longevity properties of lactobacilli and bifidobacteria [12,18,19,20], while the antimicrobial genes were involved in the anti-*Candida* effect [21]. In comparison with the *E. coli* OP50 control condition, we noted that Lcr35^®^ induced an overexpression of *daf-16* (*p* = 0.0004) and had no effect on *daf-2* (*p* = 0.9459), while *C. albicans* tended to induce an up regulation of *daf-2* (*p* = 0.0922) and a down-regulation of *daf-16* (*p* = 0.4959). In the case of *C. albicans* infection followed by a treatment with *E. coli* OP50, we noted that *daf-2* and *daf-16* genes tended to be downregulated. Conversely, with a Lcr35^®^ curative treatment, *daf-2* tended to be repressed while no change in *daf-16* expression was measured. The expression of the *sek-1* and *pmk-1* immunity genes was down and upregulated in the presence of Lcr35^®^ by 0.5-fold (*p* = 0.8679) and 3.05-fold (*p* = 0.3741), respectively, while they were up regulated by *C. albicans* 4.57-fold (*p* = 0.2038) and 4.66-fold (*p* = 0.0933), respectively. Regarding the genes of the p38 MAPK pathway, we noted no change in gene expression after infection followed by *E. coli* OP50 administration for *sek-1* but a not significant up regulation of *pmk-1*. In contrast, after curative treatment with Lcr35^®^, *sek-1* was significantly overexpressed (*p* = 0.0033) and *pmk-1* tended to be upregulated. Finally, among the 3 antimicrobials encoding the genes tested, only the overexpression of *cnc-4* was observed in the presence of Lcr35^®^ (*p* = 0.3367). *C. albicans* also induced the overexpression of *abf-2* (*p* = 0.3097), *cnc-4* (*p* = 0.0722), and *fipr-22*/*fipr-23* (*p* = 0.0736). *cnc-4* tended to be overexpressed and *fipr-22/fipr-23* tended to be repressed when the worms were treated with *E. coli* OP50. On the other hand, *abf-2* and *fipr-22/fipr-23* were significantly overexpressed (*p* = 0.0043 and *p* = 0.0209) after a treatment with Lcr35^®^.

#### 2.4.2. Effect of Lcr35^®^ Curative Treatment on DAF-16 Nuclear Translocation

**Figure 5:**
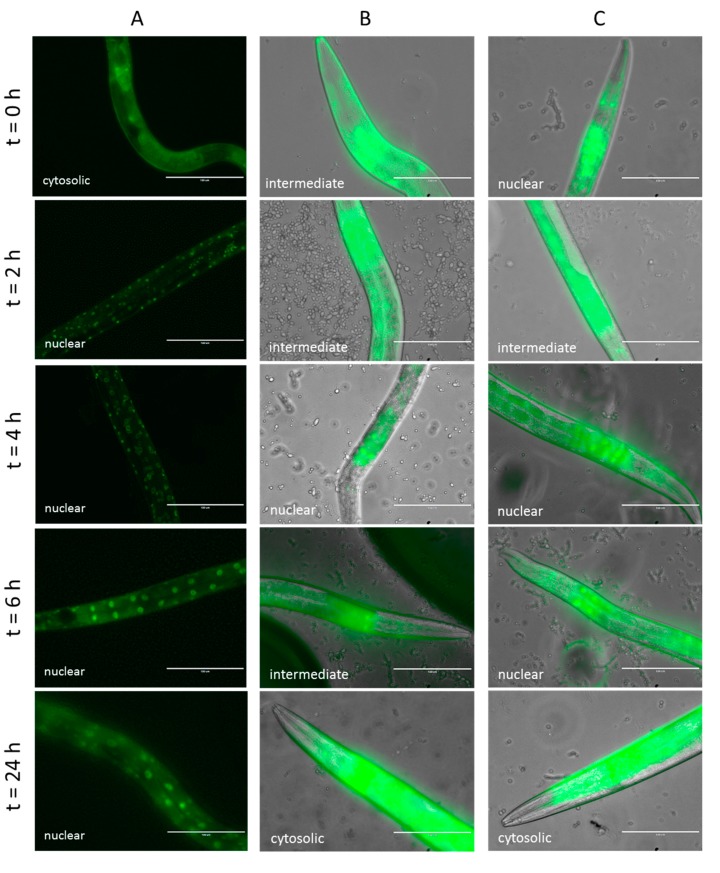
Impact of curative Lcr35^®^ treatment on DAF-16 cellular localization in *C. elegans* transgenic strain TJ-356 expressing DAF-16::GFP. Worms fed on *C. albicans* only (**A**), on *C. albicans* + OP50 (**B**), and on *C. albicans* + Lcr35^®^ (**C**). Scale bar, 100 µm.

We investigated the effect of curative treatment on the cellular localization of DAF-16 over time after infection by *C. albicans*. With our control condition (*C. albicans* only), the transcription factor DAF-16 was quickly translocated into the nuclei after only 2 h of incubation (Figure 5A). When the worms were infected by the yeast prior being fed with OP50, we observed that DAF-16 was mainly located intermediately between the cytoplasm and the nuclei during the first hours of treatment and then cytoplasmic after 24 h (Figure 5B). After a curative treatment with Lcr35^®^, the transcription factor appeared more translocated to the nuclei during the first part of the treatment before also getting back to a cytoplasmic localization (Figure 5C).

## 3. Discussion

Among the therapeutic arsenal available for the treatment of infectious diseases, including fungal diseases, LBPs appear to be a useful alternative to traditional drugs. In addition to having a very low health risk, they have broad-spectrum antimicrobial properties. Although primarily used by the population for therapeutic purposes, they were often studied as a preventive measure. Beyond the antipathogenic properties of Lcr35^®^, recent studies in humans have shown improvement in the health of patients with the administration of LBM. The effectiveness of Lcr35^®^ for the treatment of abdominal pain and intestinal functional disorders has been demonstrated in children [22,23,24] without however highlighting the molecular mechanisms. Recently, Dausset and colleagues demonstrated in women the safety and well-tolerated characteristics of a new galenic form based on Lcr35^®^, promoting the growth of endogenous vaginal *Lactobacillus*, in order to prevent an eventual dysbiosis [25]. In a previous study [12], we demonstrated that a preventive administration of Lcr35^®^ strain had two in vitro effects: inhibition of *C. albicans* growth and biofilm formation on a Caco-2 cells monolayer. Anti-*C. albicans* properties targeting in particular the adhesion of the pathogen to the epithelial cells have been attributed to exopolysaccharides (EPS) by various studies [26,27]. We then hypothesized that Lcr35^®^ may synthesize antimicrobial molecules including EPS. However, in these conditions, where Lcr35^®^ is used as a curative treatment, it no longer allows the inhibition of the pathogen on Caco-2 cells monolayers. We have performed here experiments with sequential cultures (*C. albicans* then native Lcr35^®^) on intestinal cells while Nivoliez et al. was interested in co-cultures (*C. albicans* and industrially formulated Lcr35^®^) on vaginal cells. The experimental conditions (culture medium, cells) as well as the origin of the LBM strain (native strain vs industrially formulated strain) are two parameters to take into consideration and which are very probably the cause of the difference observed compared with a previous study [11]. The mechanisms involved in a preventive context would therefore not be effective in a curative one, when the pathogen is already present on the cells. Several hypotheses could explain these results. Antimicrobial molecules that inhibit the growth of yeast may only act on dividing cells. Therefore, when the yeast is on stationary phase or does not divide, it is insensitive to these molecules. With regard to adhesion, we can wonder if *C. albicans* adheres to epithelial cell surfaces covalently or using low energy bonds. EPS secreted by Lcr35^®^ will not be able to break covalent bonds because of the absence of hydrolytic activity. If they are low energies, the competition for cell receptors may be in favor of the pathogen. The competition hypothesis between the LBM and the yeast could be further investigated by limiting the initial amount of yeast in order to favor the LBM. However, to fully understand the LBM mechanisms of action, in vitro approaches are too limited. Moving to an in vivo approach is mandatory to study the interactions between microorganisms (LBMs and pathogens) and the host response.

We choose *C. elegans* as a relevant in vivo model for studying the pathogenicity of microorganisms [21,28,29,30,31] and the antimicrobial properties of lactic acid bacteria [32,33]. Unlike in vitro experiments which demonstrated no significant antifungal effects of the LBM, those performed in vivo showed that Lcr35^®^ had interesting curative anti-*C. albicans* properties in the nematode. Contradictory results can be explained by the experimental conditions. Thus, in vitro, Lcr35^®^ is probably not in optimal conditions to allow inhibition of the yeast. This reveals the importance of using complementary experimental models (in vitro and in vivo) to overcome their limitations. During our in vivo tests, Lcr35^®^ allowed a statistically significant increase in the survival of the host, including after being contaminated by the yeast. The results obtained are in accordance with those using Lcr35^®^ in a preventive approach [12]. However, the experimental conditions are different. In a preventive approach, Lcr35^®^ has an action on its host before having an antipathogenic activity targeting *C. albicans*. In a curative approach, the LBM first has an action against yeast and eventually on its host. Sharma et al. recently tested the inhibitory properties of *Lactobacillus* spp against infection with pathogenic *E. coli* strains, by co-infection, preventive, and curative approaches. The authors have put forward several hypotheses that can explain the healing properties: the steric exclusion of the pathogen by the probiotic strains as well as the competition for the cellular receptors because of the shared carbohydrate-binding specificities with some entero-pathogens [17]. Our data showed that using supernatants did not allow optimal protection of the host, confirming the essential presence of the LBM Lcr35^®^ cells to observe the anti-*Candida* effect. Thus, it is likely that the mechanism of action is based on a mischaracterized direct interaction between Lcr35^®^ and the yeast. In the absence of pathogenic yeast, with 30–50% supernatant, we observed a significant increase in mean lifespan and worm longevity. On the other hand, during a fungal infection, only 30% of supernatant at pH = 4.5 allows an increase in mean lifespan. We hypothesize that there are two different mechanisms, one or more molecule present in the culture supernatant have a pro-longevity action while others appear to have antifungal effect. In their work, Dausset et al. demonstrated in vitro that the molecule secreted by Lcr35^®^ and having an anti *C. albicans* activity had a molecular weight smaller than 10 kDa and was resistant to protease, lipase and thermal treatments [34]. However, the molecule in question has not been identified precisely. It is conceivable that this molecule is a bacteriocin but it would be interesting to study its antifungal activity in *C. elegans*. Indeed, a recent study has shown that the bacteriocin EntV secreted by *Enterococcus faecalis* inhibited the growth of yeast and protected the nematode from infection at the same time [34,35]. In addition, another study showed the involvement of extracellular vesicles of *L. plantarum* in the activation of the host immune system against vancomycin-resistant enterococcal infection [36].

In our study, we observed that the duration of the Lcr35^®^ treatment influences the curative anti-*C. albicans* effect on nematode lifespan, suggesting that the quantity of Lcr35^®^ ingested and/or the treatment duration may have an impact on the effectiveness of the treatment. It is conceivable that there is also a dose effect explaining the results obtained with the culture supernatants. The work of Komura et al. showed that there was a dose effect linked to the pro-longevity effects of bifidobacteria, in favor of a low *Bifidobacterium*/*E. coli* OP50 ratio [37]. A thorough transcriptional study will be interesting to characterize the dose-dependent effect of LBPs administered. Therefore, we can question the relevance of the evaluation of toxicokinetic and pharmacodynamic parameters as is the case for traditional drugs. Such parameters would make it possible to adapt the prescription of LBPs and improve their effectiveness.

We demonstrated that Lcr35^®^ induced a transcriptional response in the host by activating the transcription factor DAF-16 (nuclear translocation), and the activation of the p38 MAPK signalling pathway involved genes, including in the presence of *C. albicans*. From a mechanistic point of view, several hypotheses can explain the anti-*C. albicans* properties of Lcr35^®^ in the nematode: a direct interaction between the two microorganisms as well as an immunomodulation of the host by the LBM. We have shown that even after curative treatment with the LBM, the digestive tract of the nematode is colonized by the pathogen without showing a pathological state. These data are in accordance with De Barros et al. and suggest that Lcr35^®^ induced repression of virulence factors in *C. albicans* [16]. In *C. elegans*, DAF-16 is closely related to mammalian FOXO3a, a transcription factor involved in the inflammatory process [38]. Therefore, nuclear translocation of DAF-16 by Lcr35^®^ can be interpreted as the establishment of an inflammatory response in the host allowing it to survive an infection. The work of Pukkila-Worley et al. [21] demonstrated that *C. albicans* induced a fast antifungal response in the host with the overexpression of antimicrobial genes such as *abf-2*, *cnc-4*, *cnc-7*, *fipr-22,* and *fipr-23*. With the exception of *abf-2*, all these genes are under the control of PMK-1, whose inactivation makes the nematode susceptible to infection. In our study, we showed that Lcr35^®^ curative treatment induced an up regulation of most targeted genes (*sek-1*, *pmk-1*, *fipr-22,* and *fipr-23)*, while *cnc-4* and *daf-16* remained unchanged compared to the control condition. Based on the data of Pukkila-Worley et al. [21], the presence of Lcr35^®^ would allow activation of the host’s immune defenses via the highly conserved p38 MAPK signaling pathway. Also, it is possible that the bacterium exerts a direct action on the yeast by at least partially inhibiting its virulence, thus limiting the deleterious effects of *C. albicans* in *C. elegans*. The use of *C. elegans* mutants or RNAi could be further considered deciphering with precision the signalling pathway(s) involved and the regulation mechanisms.

## 4. Materials and Methods

### 4.1. Microbial Strains and Growth Conditions

The *E. coli* OP50 strain was provided by the *Caenorhabditis* Genetics Center (Minneapolis, MN, USA) and was grown on Lysogeny Broth (LB, Miller’s Modification) (Conda, Madrid, Spain) at 37 °C overnight. The *L. rhamnosus* Lcr35^®^ strain was provided by biose^®^ (Aurillac, France) and was grown in de Man, Rogosa, Sharpe (MRS) broth (bioMérieux, Marcy l’Etoile, France) at 37 °C overnight. *C. albicans* ATCC^®^ 10231™ was grown in yeast peptone glucose (YPG) broth pH 6.5 (per L: 10 g yeast extract (Fisher Scientific, Hampton, NH, USA), 10 g peptone (Conda), 20 g glucose (Sigma, Saint-Louis, USA)) at 37 °C for 48 h. Microbial suspensions were spun down for 2 min at 1500 rpm (Rotofix 32A, Hettich Zentrifugen, Tuttlingen, Germany) and washed with M9 buffer (per L: 3 g KH_2_PO_4_, 6 g Na_2_HPO_4_, 5 g NaCl, 1 mL 1 M MgSO_4_) (Sigma) to obtain a final concentration of 100 mg/mL.

### 4.2. Influence of L. rhamnosus Lcr35^®^ on Growth of C. albicans on Caco-2 Cells Monolayer and on Biofilm Formation

Growth inhibition of *C. albicans* by the LBM Lcr35^®^ was examined using the human colorectal adenocarcinoma cell line Caco-2 [39]. Caco-2 cells were grown in Dulbecco’s modified Eagle’s minimal essential medium (DMEM, Life Technologie, Villebon-sur-Yvette, France) supplemented with 20% inactivated fetal calf serum (Life Technologie) at 37 °C with a 5% CO_2_ in air atmosphere. For the assays, the cells were seeded at a concentration of 3.5 × 10^5^ cells/well in 24-well plates (Dutscher, Brumath, France) and placed in growth conditions for 24 h. Microbial strains were grown according to Nivoliez et al. [11]. After growth, cell culture medium was removed and replaced by 1 mL of DMEM and 250 µL of *C. albicans* culture (10^7^ CFU/mL) in each well and incubated for 24 h. Lcr35^®^ (250 µL of culture (10^8^ CFU/mL)) was added in each well and incubated for 48 h. The inhibition of *C. albicans* by Lcr35^®^ was evaluated during (24 h) and after co-incubation (48 h). One hundred microliters of suspension were taken from each of the wells and the number of viable bacteria and/or yeasts was determined by plating serial dilutions of the suspensions onto MRS or Sabouraud agar plates. For the measurement of *C. albicans* biofilm formation, after incubation for 48 h, the wells were washed twice with 0.5 mL of PBS and cells harvested with 1 mL of trypsin at 37 °C. As for the inhibition assay, the number of viable bacteria or/and yeasts were determined by plating serial dilutions of the suspensions onto MRS or Sabouraud agar plates. The plates were incubated at 37 °C for 72 h (MRS) or 48 h (Sabouraud). Each assay, performed three times independently, contained two technical replicates.

### 4.3. C. elegans Maintenance

*C. elegans* N2 (wild-type) and TJ356 (*daf-16p::daf-16a/b::GFP* + *rol-6(su1006)*) strains were acquired from the *Caenorhabditis* Genetics Center. The nematodes were grown and maintained at 20 °C on nematode growth medium (NGM) (per L: 3 g NaCl (Sigma); 2.5 g peptone (Conda); 17 g agar (Biokar Diagnostics, Beauvais, France); 5 mg cholesterol (Sigma); 1 mM CaCl_2_ (Sigma); 1 mM MgSO_4_ (Sigma), 25 mL 1 M potassium phosphate (Sigma) buffer at pH 6) plates supplemented with yeast extract (4 g/L) (NGMY) and seeded with *E. coli* OP50 [40]. For all experiments, wild-type *C. elegans* N2 was used except for the study of the localization of DAF-16 (TJ356 strain).

### 4.4. C. elegans Synchronization

To avoid variations in results due to age differences, a worm synchronous population was required. Gravid worms were washed off using M9 buffer and spun down for 2 min at 1500 rpm. Five millilitres of worm bleach (2.5 mL of M9 buffer, 1.5 mL of bleach, 1 mL of 5 M sodium hydroxide) were added to the pellet and vigorously shaken until adult worm body disruption. The action of worm bleach was stopped by adding 20 mL of M9 buffer. The egg suspension was then spun down for 2 min at 1500 rpm and washed twice with 20 mL of M9 buffer. Eggs were allowed to hatch under slow agitation at 25 °C for 24 h in approximately 20 mL of M9 buffer. L1 larvae were then transferred onto NGMY plates seeded with *E. coli* OP50 until they reached the L4/young adult stage.

### 4.5. Effects of L. rhamnosus Lcr35^®^ on Candidiasis in C. elegans

Sequential feeding with Lcr35^®^ and *C. albicans* was induced in *C. elegans* in all experiments (curative assays). As control groups, a monotypic contamination was induced in *C. elegans* by inoculating with only *C. albicans*, Lcr35^®^ or *E. coli* OP50.

#### 4.5.1. Preparation of Plates Containing LBM or Pathogen Yeasts

One hundred microliters of Lcr35^®^ or *E. coli* OP50 suspension (100 mg/mL) were spread on NGMY + 0.12 mM FUdR (Sigma) plates (in order to prevent *C. elegans* progeny) and incubated at 37 °C overnight. Concerning *C. albicans* strain, 100 µL of suspension were spread on Brain Heart Infusion BHI (Biokar Diagnostics) + 0.12 mM FUdR plates and incubated at 37 °C overnight.

#### 4.5.2. Survival Assay: Curative Treatment

The survival assay was performed according to Poupet et al. [12], with some modifications. During a curative treatment, young L4 adult worms were placed on plates containing *C. albicans* for 2 h at 20 °C. Next, worms were washed with M9 buffer to remove yeasts prior being placed on Lcr35^®^, at 20 °C for different times (2, 4, 6, and 24 h). Infected nematodes were washed off plates using M9 buffer prior to be transferred into a 6-well microtiter plate (approximately 50 worms per well) containing 2 mL of BHI/M9 (20%/80%) + 0.12 mM FUdR liquid assay medium per well and incubated at 20 °C. For the control groups (i.e., *C. albicans* + *E. coli* OP50, *E. coli* OP50 alone, Lcr35^®^ alone and *C. albicans* alone), worms were treated in the same way. Nematodes were observed daily and were considered dead when they did not respond to a gentle mechanical stimulation. This assay was performed as three independent experiments containing three wells per condition.

### 4.6. Study of L. rhamnosus Lcr35^®^ Cell-Free Supernatant on Candidiasis in C. elegans

The potential protective effect of Lcr35^®^ cell-free supernatant was investigated. For this, a culture of Lcr35^®^ was carried out in MRS broth and incubated for 24 h at 37 °C. The broth was centrifuged for 15 min at 14,000 rpm (Rotofix 32A, Hettich Zentrifugen). The supernatant was then sterilized using a 0.22 μm filter. As before, L4/young adult worms were placed on plates containing *C. albicans* for 2 h at 20 °C. Next, worms were washed with M9 buffer to remove yeasts prior being transferred into a 6-well microtiter plate (about 50 worms per well) containing 2 mL of BHI/M9 (20%/80%) liquid assay medium supplemented by Lcr35^®^ cell-free supernatant (30%, 50%, 100% at pH = 4.5 or pH = 7) and 0.12 mM FUdR per well and incubated at 20 °C. Regarding the supernatant at pH = 7, it was neutralized with a 5N sodium hydroxide solution (Panreac, Barcelona, Spain). For the control groups (i.e., *E. coli* OP50 + Lcr35^®^ cell-free supernatant), worms were treated in the same way. Nematodes were observed daily and were considered dead when they did not respond to a gentle mechanical stimulation. This assay was performed as three independent experiments containing three wells per condition.

### 4.7. Visualization of C. albicans in C. elegans Intestine

In order to study the presence of the pathogen *C. albicans* in the worm gut, a fluorescent staining of the yeast was performed. The yeast was stained with DAPI (Thermo Scientific, Karlsruhe, Germany) according to the manufacturer’s instructions. A fresh culture of *C. albicans* was performed in YPG broth as described in Section 4.1, 10 μL of DAPI at 300 nM was added to 1 mL of *C. albicans* suspension and incubated at room temperature in the dark for 15 min. The unbound dye was removed by centrifugation (14,000 rpm for 5 min at 4 °C) (Beckman J2-MC Centrifuge, Beckman Coulter, Brea, USA) and washed with 1 mL of M9 buffer. Subsequently, the nematodes were fed on labelled-*C. albicans* on BHI plates for 72 h and then with *E. coli* OP50 or Lcr35^®^ on NGMY plates for 24 h. The nematodes were then visualized using a fluorescence microscope at 100× magnification (Evos FL, Invitrogen).

### 4.8. RNA Isolation and Real-Time Quantitative PCR

Approximately 10,000 worms were harvested from NGMY plates with M9 buffer. Total RNA was extracted by adding 500 µL of TRIzol reagent (Ambion by Life Technologies, Carlsbad, USA). Worms were disrupted using a Precellys (Bertin Instruments, Montigny-le-Bretonneux, France) and glass beads (PowerBead Tubes Glass 0.1 mm, Mo Bio Laboratories, USA). Beads were removed by centrifugation at 14,000 rpm for 1 min (Eppendorf^®^ 5415D, Hamburg, Germany), and 100 µL of chloroform were added to the supernatant. Tubes were vortexed for 30 s and incubated at room temperature for 3 min. The phenolic phase was removed by centrifugation at 12,000 rpm for 15 min at 4 °C. The aqueous phase was treated with chloroform as previously described. RNA was precipitated by adding 250 µL of isopropanol for 4 min at room temperature and spun down at 12,000 rpm for 10 min (4 °C). The supernatant was discarded, and the pellet was washed with 1 mL of 70% ethanol. The supernatant was discarded after centrifugation at 14,000 rpm for 5 min (4 °C), and the pellet was dissolved in 20 µL of RNase-free water. RNA was reverse-transcribed using a High-Capacity cDNA Archive kit (Applied Biosystems, Foster City, USA) according to the manufacturer’s instructions. For real-time qPCR assay, each tube contained 2.5 µL of cDNA, 6.25 µL of Rotor-Gene SYBR Green Mix (Qiagen GmbH, Hilden, Germany), 1.25 µL of 10 µM primers (reported in Table 3) (Eurogentec, Seraing, Belgium), and 1.25 µL of water. All samples were run in triplicate. Rotor-Gene Q Series Software (Qiagen GmbH) was used for the analysis. The quantification of gene-of-interest expression (E_GOI_) was performed according to the following formula [41] taking into account the efficiency of the PCR for each primer pair and normalizing the expression of the gene of interest by two reference genes: *cdc-42* and Y45F10D.4.

(1) $$E_{\mathrm{GOI}}=\frac{{\left(\mathrm{GOI}\;\mathrm{efficiency}\right)}^{{\Delta \mathrm{Ct}}_{\mathrm{GOI}}}}{\sqrt{{\left(cdc-42\;\mathrm{efficiency}\right)}^{{\Delta \mathrm{Ct}}_{cdc-42}}\times {\left(Y45F10D.4\;\mathrm{efficiency}\right)}^{{\Delta \mathrm{Ct}}_{Y45F10D.5}}}}$$

The worms fed with *E. coli* OP50 were used as control conditions for the gene expression calculation.

### 4.9. DAF-16 Nuclear Localization

DAF-16 nuclear localization was followed as described by others [45] using a transgenic TJ-356 worm strain constitutively expressing the DAF-16 transcription factor fused to GFP (DAF-16::GFP). Once adults, worms were exposed to *C. albicans* for 2, 4, 6, and 24 h at 20 °C. A curative approach was conducted: worms were put in the presence of *C. albicans* for 2 h and after with *E. coli* OP50 or Lcr35^®^ for 4 h at 20 °C. The nematodes were subsequently imaged 2, 4, 6, and 24 h after infection. The translocation of DAF-16::GFP was scored by the observation of the presence of GFP accumulation in the *C. elegans* cell nuclei, using a fluorescence microscope at 40× magnification (Evos FL, Invitrogen).

### 4.10. Statistical Analysis

Data are expressed as the mean ± standard deviation. The *C. elegans* survival assay was examined by using the Kaplan-Meier method, and differences were determined by using the log-rank test with R software version 3.6.1 [46], and the survival [47] and survminer [48] packages. For *C. albicans* growth inhibition and *C. elegans* gene expressions, differences between conditions were determined by a two-way ANOVA followed by a Fisher’s Least Significant Difference (LSD) post hoc test using GraphPad Prism version 8.2.0 for macOS (GraphPad Software, La Jolla, California, USA). For *C. albicans* biofilm formation, differences between conditions were determined by an unpaired t test using GraphPad Prism version 8.2.0 for macOS. Each experiment was performed in three different replicates. A difference with *p*-value ≤ 0.05 was considered as significant.

## 5. Conclusions

This study showed the effectiveness of the LBM Lcr35^®^ in a curative context. Although it did not allow the reduction of the fungal growth in vitro in our experimental conditions, it had in vivo antifungal capabilities with a protection of the *C. albicans* infected worm *C. elegans*. Our data suggest that a Lcr35^®^ treatment tends to activate *C. elegans* immune response (overexpression genes of the p38 MAPK pathway and encoding for antimicrobials). An exhaustive transcriptomic study would allow a better understanding of the interactions between *C. elegans*, *C. albicans* and Lcr35^®^. Once the molecular mechanisms well characterized, it would be of interest to evaluate the possibility to extrapolate to other strains of *Lactobacillus* spp. and *Candida* spp. and to include the *C. elegans* approaches in the usual tests allowing the identification of new live biotherapeutic microorganisms.

## Figures and Tables

**Table 1:** Effects of Lcr35^®^ cell-free supernatant on *C. elegans* survival, worms previously fed on *E. coli* OP50 or *C. albicans*. Log rank test was performed with *E. coli* OP50 without Lcr35^®^ supernatant as a control.

Microorganism	Lcr35^®^ Supernatant Concentration (%)	Lcr35^®^ Supernatant pH	*C. elegans* Mean Lifespan (days)	*C. elegans* Longevity (days)	Log Rank Test (*p*-Value)
*E. coli* OP50	no supernatant		9	14	N/A
Lcr35^®^	no supernatant		13	17	<2.10^–16^
*C. albicans*	no supernatant		4	9	<2.10^–16^
*E. coli* OP50	30	4,5	15	20	<2.10^–16^
	30	7	13	18	<2.10^–16^
	50	4,5	12	15	2.10^–9^
	50	7	14	21	<2.10^–16^
	100	4,5	1	8	<2.10^–16^
	100	7	1	8	<2.10^–16^
*C. albicans*	30	4,5	5	6	<2.10^–16^
	30	7	2	3	<2.10^–16^
	50	4,5	4	8	<2.10^–16^
	50	7	2	3	<2.10^–16^
	100	4,5	1	5	<2.10^–16^
	100	7	2	3	<2.10^–16^

**Table 2:** Relative expression of *C. elegans* genes of interest in presence of Lcr35^®^ and *C. albicans* in pure or in sequential cultures in comparison with the control condition *E. coli* OP50 (alone). Genes were considered differentially expressed when the *p*-value was lower than 0.05 (*), 0.01 (**), or 0.001 (***) according to Fisher’s LSD test, and simultaneously when the expression change was of at least 2 times or 0.5 times.

	Genes of Interest						
	Insulin Signaling Pathway		p38 MAPK Signaling Pathway		Antimicrobials		
Conditions	*daf*-*2*	*daf-16*	*sek-1*	*pmk-1*	*abf-2*	*cnc-4*	*fipr-22*/*fipr-23*
*E. coli* OP50	1	1	1	1	1	1	1
Lcr35^®^	0.97	5.84 ***	0.50	3.05	0.90	8.48	0.89
*C. albicans*	1.87	0.31	4.57	4.66	3.58	14.31	3.45
*C. albicans* + *E. coli* OP50	0.52	0.41	1.01	2.57	0.95	7.04	0.26
*C. albicans* + Lcr35^®^	0.61	1.47	11.24 **	2.42	8.00 **	1.31	4.29 *

**Table 3:** Targeted *C. elegans* genes primers for qPCR analysis.

Gene Name	Gene Type	Forward Primer (5′–3′)	Reverse Primer (5′–3′)	Reference
*cdc-42*	housekeeping	ATCCACAGACCGACGTGTTT	GTCTTTGAGCAATGATGCGA	[42]
Y45F10D.4	housekeeping	CGAGAACCCGCGAAATGTCGGA	CGGTTGCCAGGGAAGATGAGGC	[43]
*daf-2*	GOI	AAAAGATTTGGCTGGTCAGAGA	TTTCAGTACAAATGAGATTGTCAGC	[44]
*daf-16*	GOI	TTCAATGCAAGGAGCATTTG	AGCTGGAGAAACACGAGACG	[44]
*sek-1*	GOI	GCCGATGGAAAGTGGTTTTA	TAAACGGCATCGCCAATAAT	[44]
*pmk-1*	GOI	CCGACTCCACGAGAAGGATA	AGCGAGTACATTCAGCAGCA	[44]
*abf-2*	GOI	TCGTCCGTTCCCTTTTCCTT	CCTCTCTTAATAAGAGCACC	[12]
*fipr-22/fipr-23*	GOI	CCCAATCCAGTATGAAGTTG	ATTTCAGTCTTCACACCGGA	[12]
*cnc-4*	GOI	ATGCTTCGCTACATTCTCGT	TTACTTTCCAATGAGCATTC	[12]
*cnc-7*	GOI	TTTTGTTGGCTCTGGTGGCA	ATGAGTCCAGGACGGTACAT	[12]
